# Perioperative Management of One-Lung Ventilation in an Adolescent With Metastatic Osteosarcoma Complicated by Bilateral Pneumothoraces

**DOI:** 10.7759/cureus.96738

**Published:** 2025-11-13

**Authors:** Sheena Mary Boban, Milind B Thakur, Ali Rasmi Ibrahim, Sanaa Mohamed Ahmed Ghali, Rajesh Pattanayak

**Affiliations:** 1 Anesthesiology, American Hospital, Dubai, ARE

**Keywords:** anesthesia, bilateral pneumothorax, olv, one-lung ventilation, osteosarcoma, pediatric thoracic surgery, vats

## Abstract

Thoracic anesthesia in pediatric patients is challenging due to anatomical and physiological factors that increase susceptibility to hypoxemia during one-lung ventilation (OLV). These complexities are further magnified in children with underlying oncological disease and poor pulmonary reserve. We report anesthetic management of a 16-year-old, 32 kg boy with metastatic osteosarcoma presenting with recurrent bilateral pneumothoraces scheduled for right video-assisted thoracoscopic surgery (VATS) and pleurodesis. The coexistence of bilateral pneumothoraces with compromised pulmonary reserve, with a potential risk of tension pneumothorax, posed significant anesthetic challenges, particularly for OLV. We describe perioperative considerations, anesthetic technique, and strategies to maintain oxygenation and hemodynamic stability. This case highlights the importance of careful planning, lung-protective ventilation, and multidisciplinary coordination in managing pediatric thoracic cases.

Anesthetic management included standard American Society of Anesthesiologists (ASA) monitoring with invasive arterial pressure, induction with remifentanil, propofol, and rocuronium, and placement of a left-sided double-lumen tube, confirmed with fiberoptic bronchoscopy. A lung-protective strategy was applied with low tidal volumes, titration of fraction of inspired oxygen (FiO_2_), stepwise positive end-expiratory pressure (PEEP) adjustments, and tolerance of permissive hypercapnia. Due to the risk of desaturation, intraoperative SpO_2_ was maintained between 92% and 95% through FiO_2_ adjustments, recruitment maneuvers, and judicious hemodynamic support with ephedrine. Postoperatively, after confirming successful reinflation of the right lung, the patient was successfully extubated, managed with multimodal intravenous analgesia, and had a stable recovery by day three. This case underscores the importance of meticulous anesthetic planning, vigilant intraoperative management, and multimodal analgesia in pediatric oncology patients undergoing thoracic surgery. Tailored strategies for OLV in children with bilateral pneumothoraces and metastatic disease are critical to ensure safe outcomes.

## Introduction

Anesthesia for thoracic surgeries in pediatric patients is inherently challenging due to anatomical, physiological, and technical considerations. Unlike adults, children have smaller airways, reduced functional residual capacity, and increased oxygen consumption, rendering them vulnerable to rapid desaturation during one-lung ventilation (OLV) [1]. Video-assisted thoracoscopic surgery (VATS) is increasingly employed for diagnostic and therapeutic interventions in pediatric patients, necessitating advanced anesthetic techniques for lung isolation and safe perioperative management [2].

One-lung ventilation is technically difficult in children because of limited airway sizes and fewer options for lung isolation devices [3]. While bronchial blockers and modified endotracheal tubes are alternatives, double-lumen tubes (DLTs) remain the most reliable method in adolescents weighing more than 30 kg [4]. Maintaining oxygenation during OLV requires strategies such as lung-protective ventilation, FiO_2_ titration, application of PEEP, and tolerance of permissive hypercapnia [5].

These challenges become even more pronounced in children with oncological disease, particularly metastatic osteosarcoma with pulmonary involvement. Osteosarcoma, the most common primary malignant bone tumor in children and adolescents, frequently metastasizes to the lungs, resulting in recurrent pneumothoraces and impaired respiratory reserve [6,7]. Surgical intervention in these patients often involves VATS wedge resection or pleurodesis for recurrent air leaks, necessitating careful anesthetic planning [8].

In addition, chemotherapy-induced cytopenias, nutritional compromise, and prior thoracic procedures increase perioperative risk [9]. Pain management is also complex; while thoracic epidural analgesia is standard in thoracotomy, it may be contraindicated in patients with deranged coagulation or immunosuppression [10]. Hence, multimodal systemic analgesia becomes a preferred strategy [11].

In this context, we present a case of a 16-year-old boy with advanced metastatic osteosarcoma and bilateral persistent pneumothoraces who underwent right-sided VATS pleurodesis under OLV. The report highlights the anesthetic challenges of managing OLV in a fragile pediatric oncology patient with compromised lung function and describes strategies to optimize intraoperative oxygenation and postoperative recovery.

## Case presentation

A 16-year-old boy, weighing 32 kg, with a history of metastatic osteosarcoma of the left distal femur, was admitted with recurrent bilateral spontaneous pneumothoraces. His oncological history included initial diagnosis in 2020, treated with multi-agent chemotherapy, surgical resection of the distal femur with prosthetic reconstruction, and mifamurtide therapy, achieving >90% tumor necrosis. Despite initial remission, he developed multiple relapses. In March 2024, recurrent osteosarcoma with pulmonary and osseous metastases was confirmed by lung biopsy, and he underwent further chemotherapy until September 2024. In November 2024, disease progression occurred with relapse involving the femur, spine, pelvis, and liver. He underwent hepatic artery embolization in July 2025 and remained on palliative chemotherapy with cabozantinib and radiotherapy for chest wall metastases. Over the course of his illness, he had multiple admissions for bilateral pneumothoraces requiring intercostal chest drains, including a right lower lobectomy in 2024 for a pulmonary nodule.

At presentation, he was pale, emaciated, and mildly dyspneic on exertion, with a baseline SpO_2_ of 95% on room air. Chest auscultation revealed bilateral diminished air entry, more pronounced on the right side. Cardiovascular and neurological examinations were normal. He was classified as ASA physical status III.

Preoperative evaluation

The patient reported chest discomfort but was otherwise comfortable and communicative. Due to his fragile condition, preoperative optimization focused on stabilization, cross-matching of blood products, and coordination with cardiothoracic surgery and oncology teams. Anesthesia consent was obtained after explaining risks of hypoxemia, need for OLV, and possible postoperative ventilatory support. Table 1 presents the preoperative laboratory findings, which revealed mild anemia (hemoglobin: 10.2 g/dL), chemotherapy-induced leukopenia (WBC: 3.6×10^9^/L), and lymphopenia (8.1%), indicating immunosuppression, while platelet count (248×10^9^/L) and coagulation profile were within normal limits, ensuring adequate hemostatic function for surgery.

**Table 1:** Preoperative laboratory findings.

Parameters	Values	Reference range
Hemoglobin	10.2 g/dL	13-17 g/dL
WBC	3.6×10^9^/L	4-10×10^9^/L
Platelets	248×10^9^/L	150-410×10^9^/L
Neutrophils	73%	40-80%
Lymphocytes	8.1%	20-40%
Prothrombin time	13.2	11-15 s
INR	1.03	0.9-1.2

**Figure 1:**
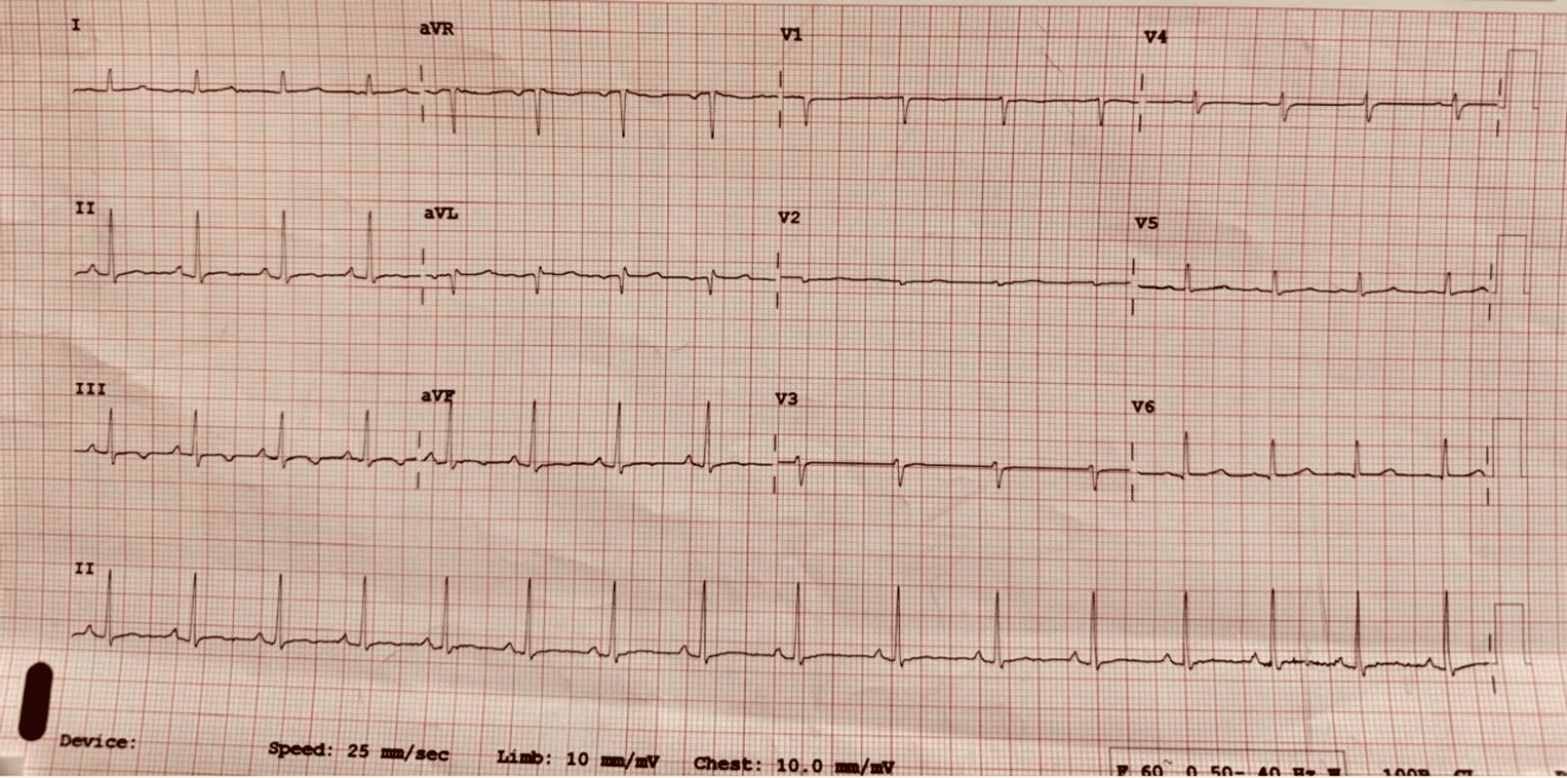
ECG with low voltage complexes.

Figure 1 shows an ECG that revealed low-voltage complexes in the precordial leads and a normal sinus rhythm at approximately 95 beats per minute, with normal P-wave morphology, PR interval, QRS duration, and QTc interval. There were no ST-segment or T-wave abnormalities.

**Figure 2:**
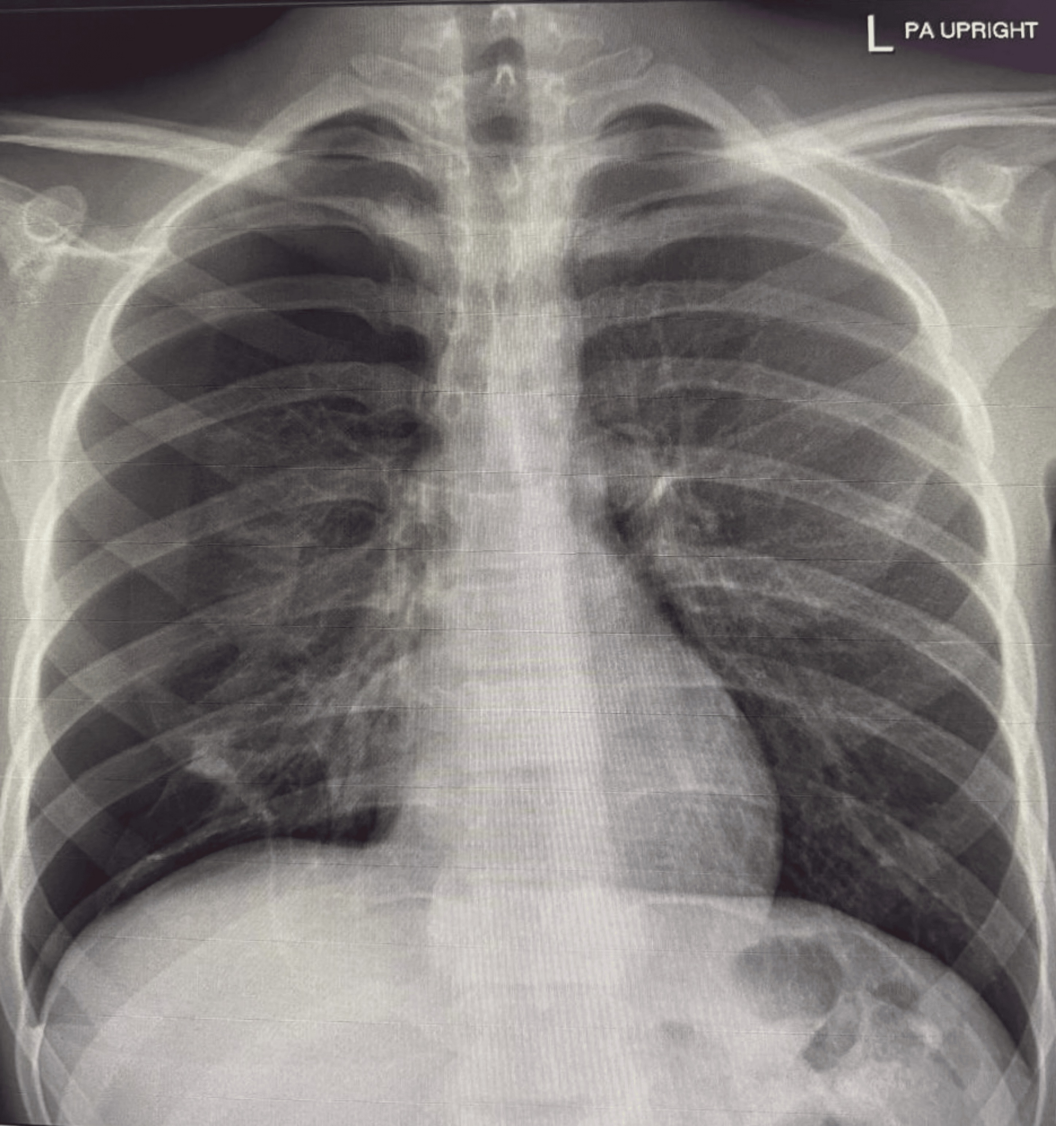
Chest X-ray posteroanterior (PA) view.

**Figure 3:**
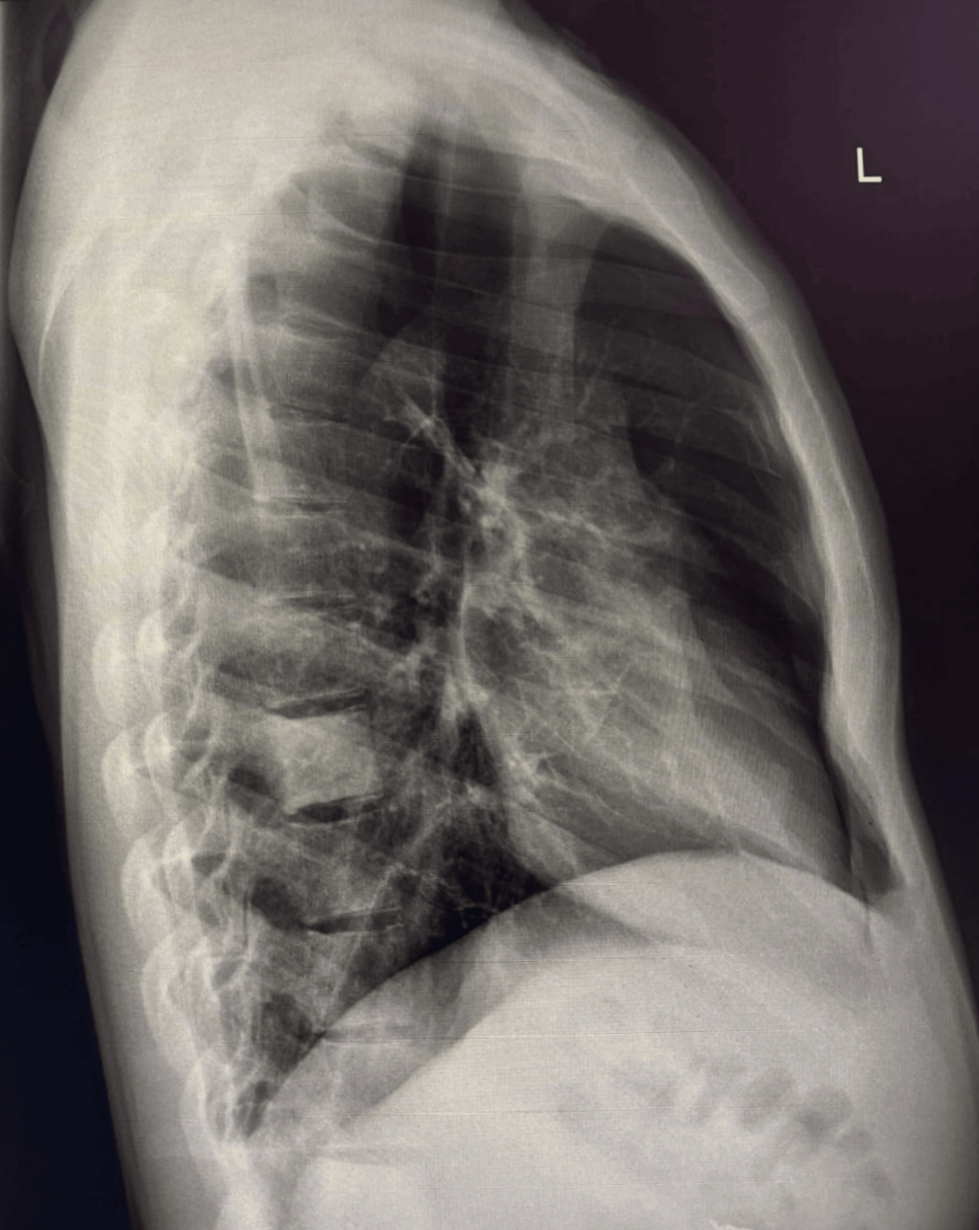
Chest X-ray lateral view.

Chest X-ray posteroanterior/PA view and lateral view demonstrated a persistent right-sided pneumothorax, evidenced by a visible pleural line with absence of peripheral lung markings, and associated collapse of the right lung parenchyma (Figures 2, 3).

Intraoperative anesthetic management

Monitoring included ECG, SpO_2_, non-invasive blood pressure, invasive arterial line, and capnography. After preoxygenation, anesthesia was induced with remifentanil target-controlled infusion (TCI) at 0.1 μg/kg/min, propofol 2-3 mg/kg (~60 mg), and rocuronium 20 mg. A 35 Fr left-sided double-lumen tube was inserted under direct laryngoscopy and confirmed by fiberoptic bronchoscopy (Table 2).

**Table 2:** Intraoperative anesthetic management. TCI: target-controlled infusion

Variable	Details
Airway device	35 Fr left-sided double-lumen tube (DLT)
Induction agent	Remifentanil TCI, propofol, rocuronium
Ventilation strategy	Lung-protective OLV, permissive hypercapnia
Hemodynamics	Supported with crystalloids and ephedrine bolus
Analgesia	IV paracetamol, oxycodone
Surgical approach	Right VATS via 3 ports
Intraoperative findings	Multiple metastatic white nodules, bullae
Procedure performed	Wedge resection with pleural irrigation and talc pleurodesis
Drains	24F bilateral chest drains with talc instillation
Blood loss	Minimal
Intraoperative events	Uneventful

The patient was ventilated using a lung-protective OLV strategy with tidal volumes of 6-7 mL/kg (192-224 mL), an FiO_2_ of 0.6-0.8, and a PEEP of 5 cm H_2_O. Permissive hypercapnia was allowed, which involves deliberately allowing a moderate elevation of PaCO_2_ (typically 45-60 mmHg) to reduce airway pressures and minimize barotrauma. The right lung was deflated for VATS. During OLV, intermittent desaturation occurred. Expansion of the dependent lung to improve oxygenation was limited because surgical exposure required the lung to remain collapsed. Oxygenation was maintained through FiO_2_ adjustments, permissive hypercapnia, and intermittent recruitment maneuvers as feasible, while careful communication with the surgical team ensured that any necessary lung inflation did not compromise the procedure. Hemodynamic stability was maintained with crystalloids and ephedrine when needed. Analgesia consisted of IV paracetamol 15 mg/kg (~480 mg) and oxycodone. A thoracic epidural was avoided due to chemotherapy-related cytopenia risks. Local anesthetic was given at the port sites.

After induction of general anesthesia and establishment of one-lung ventilation, the patient was positioned in the left lateral decubitus position with the right side up, and VATS ports were placed. The pleural cavity was irrigated, and the underwater test confirmed that there was no ongoing air leak. Attention was then directed to pleurodesis - parietal pleura abrasion was performed, and talc pleurodesis was administered. A 24F straight chest drain was placed under direct vision, and lung inflation was confirmed. The left-sided drain was clamped, and talc slurry (3 g) was instilled through the drain, with redistribution achieved by repositioning the patient in lateral and supine orientations. The surgical procedure lasted 90 min with minimal blood loss. No intraoperative complications occurred. At completion, bilateral lung expansion was confirmed, and airway suctioning was performed. The patient was extubated successfully after full recovery of consciousness and neuromuscular function and transferred to the PICU for postoperative monitoring with chest drains kept on suction (-20 cmH_2_O).

Postoperative course

Postoperatively, the patient was managed in the ICU with supplemental oxygen, incentive spirometry, and multimodal IV analgesia. He remained hemodynamically stable, with good lung expansion on chest radiography. Pain control was satisfactory.

## Discussion

The anesthetic management of pediatric thoracic surgery, particularly with OLV, requires careful planning and execution. OLV in adolescents is feasible with appropriately sized double-lumen tubes, which provide effective lung isolation and improved surgical exposure compared to bronchial blockers [12]. However, hypoxemia remains a common challenge, necessitating strategies such as increasing FiO_2_, applying PEEP to the dependent lung, intermittent recruitment maneuvers, and accepting permissive hypercapnia [13,14].

In our patient, the anesthetic challenges included significant frailty, the risk of developing tension pneumothorax under positive pressure ventilation, difficulties in ventilation due to compromised pulmonary reserve, and the potential for hemodynamic instability. OLV was complicated by underlying bilateral pneumothoraces, and maintaining oxygenation was paramount. The combination of FiO_2_ titration, low tidal volumes, and recruitment strategies ensured safe SpO_2_ levels throughout surgery.

Hemodynamic management is equally critical. Patients with advanced malignancy often exhibit poor nutritional status, anemia, and chemotherapy-induced marrow suppression [15]. Our patient had anemia and leukopenia, necessitating preoperative cross-matching and vigilance against infection. Low-dose ephedrine bolus was used to support systemic pressures without fluid overload, minimizing the risk of worsening pulmonary edema.

Pain management is another important consideration. Although thoracic epidural analgesia provides superior postoperative pain control, it is often contraindicated in oncology patients due to potential coagulopathy, immunosuppression, or thrombocytopenia [16]. In this case, multimodal systemic analgesia achieved satisfactory pain relief while avoiding regional block-related risks.

This case illustrates the value of multidisciplinary coordination. Collaboration between anesthesiologists, surgeons, intensivists, and oncologists ensured optimized perioperative care. Pediatric thoracic anesthesia guidelines emphasize tailoring OLV and analgesia techniques to the patient’s comorbidities, anatomy, and surgical goals [17,18].

Published reports have described safe OLV in adolescents with lung pathology, though outcomes are variable depending on disease burden [19]. Our case adds to the limited literature on OLV management in pediatric oncology with bilateral pneumothoraces, demonstrating that careful anesthetic strategies can yield favorable perioperative outcomes even in high-risk patients. Furthermore, contemporary reviews highlight the importance of structured perioperative planning, vigilant intraoperative monitoring, and multidisciplinary collaboration as essential elements in the management of pediatric thoracic surgery, reinforcing the principles demonstrated in this case [20].

## Conclusions

This case underscores significant anesthetic challenges encountered in managing a pediatric oncology patient with bilateral pneumothoraces undergoing VATS pleurodesis. Successful anesthetic management required achieving an optimal balance between maintaining adequate oxygenation during one-lung ventilation and minimizing the risks of barotrauma and hemodynamic instability. Critical elements included thorough preoperative assessment, vigilant intraoperative monitoring, lung-protective ventilation with permissive hypercapnia, and cautious hemodynamic support. Multimodal systemic analgesia was employed in preference to neuraxial techniques due to the potential risks associated with chemotherapy-induced cytopenias.

Despite advanced metastatic disease and limited pulmonary reserve, the patient tolerated anesthesia and surgery well, with stable intraoperative parameters and an uncomplicated postoperative recovery. This case highlights the importance of individualized anesthetic planning guided by physiological principles and tailored to the patient’s comorbidities. Close communication between anesthesiology, surgical, and critical care teams was pivotal in ensuring safety throughout the perioperative course. The experience reinforces that early anticipation of respiratory challenges, readiness to modify ventilatory strategies, and comprehensive postoperative care are essential to optimize outcomes. Ultimately, a multidisciplinary, patient-centered approach remains the cornerstone of successful anesthetic management in complex pediatric thoracic oncology surgery. Through this report, we aim to contribute to the growing body of knowledge guiding physicians in managing patients with metastatic cancers involving the lungs, providing practical insights that may enhance perioperative decision-making and patient safety in similar high-risk scenarios.
